# Prevalence, predictors and outcomes of self-reported feedback for EMS professionals: a mixed-methods diary study

**DOI:** 10.1186/s12873-024-01082-y

**Published:** 2024-09-13

**Authors:** Caitlin Wilson, Luke Budworth, Gillian Janes, Rebecca Lawton, Jonathan Benn

**Affiliations:** 1 School of Psychology, University of Leeds, Leeds, LS2 9JT UK; 2 Yorkshire Ambulance Service Research Institute, Yorkshire Ambulance Service NHS Trust, Wakefield, WF2 0XQ UK; 3 Yorkshire Quality and Safety Research Group, Bradford Institute for Health Research, Bradford, BD9 6RJ UK; 4 NIHR Yorkshire & Humber Patient Safety Research Collaboration, Bradford Teaching Hospitals NHS Foundation Trust, Bradford, BD9 6RJ UK; 5 Faculty of Health and Education, Manchester Metropolitan University, Manchester, M15 6BH UK; 6 Present Address: Faculty of Health, Medicine and Social Care, Anglia Ruskin University, Chelmsford, CM1 1SQ UK

**Keywords:** Feedback, Prehospital care, Emergency medical services, Professional development, Staff wellbeing, Diary methods, Multilevel modelling

## Abstract

**Background:**

Providing feedback to healthcare professionals and organisations on performance or patient outcomes may improve care quality and professional development, particularly in Emergency Medical Services (EMS) where professionals make autonomous, complex decisions and current feedback provision is limited. This study aimed to determine the content and outcomes of feedback in EMS by measuring feedback prevalence, identifying predictors of receiving feedback, categorising feedback outcomes and determining predictors of feedback efficacy.

**Methods:**

An observational mixed-methods study was used. EMS professionals delivering face-to-face patient care in the United Kingdom’s National Health Service completed a baseline survey and diary entries between March-August 2022. Diary entries were event-contingent and collected when a participant identified they had received feedback. Self-reported data were collected on feedback frequency, environment, characteristics and outcomes. Feedback environment was measured using the Feedback Environment Scale. Feedback outcomes were categorised using hierarchical cluster analysis. Multilevel logistic regression was used to assess which variables predicted feedback receipt and efficacy. Qualitative data were analysed using content analysis.

**Results:**

299 participants completed baseline surveys and 105 submitted 538 diary entries. 215 (71.9%) participants had received feedback in the last 30 days, with patient outcome feedback the most frequent (*n* = 149, 42.8%). Feedback format was predominantly verbal (*n* = 157, 73.0%) and informal (*n* = 189, 80.4%). Significant predictors for receiving feedback were a paramedic role (aOR 3.04 [1.14, 8.00]), a workplace with a positive feedback-seeking culture (aOR 1.07 [1.04, 1.10]) and white ethnicity (aOR 5.68 [1.01, 29.73]). Feedback outcomes included: personal wellbeing (closure, confidence and job satisfaction), professional development (clinical practice and knowledge) and service outcomes (patient care and patient safety). Feedback-seeking behaviour and higher scores on the Feedback Environment Scale were statistically significant predictors of feedback efficacy. Solicited feedback improved wellbeing (aOR 3.35 [1.68, 6.60]) and professional development (aOR 2.58 [1.10, 5.56]) more than unsolicited feedback.

**Conclusion:**

Feedback for EMS professionals was perceived to improve personal wellbeing, professional development and service outcomes. EMS workplaces need to develop a culture that encourages feedback-seeking to strengthen the impact of feedback for EMS professionals on clinical decision-making and staff wellbeing.

**Supplementary Information:**

The online version contains supplementary material available at 10.1186/s12873-024-01082-y.

## Background

The National Health Service (NHS) staff survey [1] consistently identifies Emergency Medical Services (EMS) professionals as the group with the highest work-related stress (55.7%), burnout (49.3%) and leaving intentions (42.9%) – with ~ 25% having applied for non-NHS jobs post COVID-19 [2]. Receiving feedback on patient outcomes and personal performance may improve job support for EMS professionals and enhance staff wellbeing, job satisfaction and patient care [3, 4].

Across healthcare settings, including EMS, clinical performance feedback has been demonstrated to improve quality of care and professional development [5, 6]. However, recent reviews of existing literature and current practice [7] recommend further research on the provision of patient outcome feedback and the impact of feedback on staff wellbeing in EMS.

EMS professionals could particularly benefit from feedback as their work environment is characterised by complexity, uncertainty and extreme stressors [8, 9]. EMS professionals work autonomously, making complex decisions including assessing and treating patients at home to avoid unnecessary hospital attendance and reduce demand on emergency departments [10, 11]. Nevertheless, providing and accessing, EMS feedback on decision-making is difficult due to constraints such as a mobile workforce, disconnected digital technology [12] and data sharing governance issues [4].

When feedback is provided for EMS professionals this is typically through formal initiatives, such as performance feedback during appraisals, patient outcome feedback from “post-box” schemes and patient-experience feedback through thank-you letters [3, 7]. However, qualitative research suggests that EMS professionals desire more and better feedback, especially concerning patient outcomes [3, 4, 13]. When formal feedback initiatives are lacking, EMS professionals informally approach ED staff seeking feedback on patient outcomes [3]. However, informal feedback is limited by patient confidentiality issues, information quality, verbal format and geographical barriers [4, 13]. While systematic reviews [6] and current practice [7] suggest formal feedback to EMS professionals positively affects patient care and clinical performance, it is unknown whether informal feedback or actively solicited feedback have similar outcomes.

In the United States (US), it is estimated that feedback is provided to EMS professionals in just 24% of encounters [14] with 50–69% of paramedics self-reporting having received feedback in the previous month [15, 16]. Particular recipient and contextual characteristics appear associated with increased feedback, including staff with higher level certifications, fewer years’ experience and working in busier or hospital-based organisations [15].

Learning more about how the context and format of feedback impacts outcomes, as well as the mechanisms through which feedback influences outcomes, could be an important step in enhancing feedback effectiveness in EMS [17]. In this vein, Clinical Performance Feedback Intervention Theory, which has good face validity in the prehospital setting [3], offers 42 hypotheses of when feedback is more effective e.g. when feeding back to staff with positive beliefs about feedback [18]. Feedback effectiveness is also predicted by the extent to which an organisation encourages, provides and uses feedback, i.e. the ‘feedback environment’ [19, 20], whereby a positive feedback environment predicts positive outcomes for individuals and organisations [21–24].

Despite increasing research interest in prehospital feedback, no studies have explored the content and outcomes of prehospital feedback prospectively, or assessed feedback prevalence and predictors amongst EMS professionals in the United Kingdom. International studies have been limited by not drawing upon existing theory and potential recall bias [15, 16]. This study aimed to address these gaps by answering the following research questions:

- How prevalent is feedback for UK EMS professionals and what types of feedback do they receive?
- What individual and contextual factors predict EMS professionals receiving feedback in the previous 30 days?
- What are the perceived outcomes of feedback for EMS professionals?
- What predicts instances of self-reported feedback being perceived as improving outcomes?

## Methods

### Study design

This observational mixed-methods study consisted of a baseline survey followed by diary entries. Collecting diary entries in real time is known to reduce recall bias by collecting data at the level of feedback events and therefore not relying on generalised reflections of feedback provision over a period of time, whilst enabling analysis of within- and between-person variability [25]. Diary entries were event-contingent and collected when a participant identified they had received feedback. Diary entries on desired feedback and a follow-up survey were part of the study but are not reported here.

This mixed-methods study followed the approach defined by Creswell and Plano Clark [26] as ‘triangulation design: quantitative data model’. The primary emphasis of data collection was quantitative survey data, which was supported by open-ended questions in the baseline survey and diary entry form to contextualise and expand upon quantitative results.

Ethical approval was granted from the University of Leeds ethics committee (PSYC-406 04/01/2022) and the Health Research Authority (ID: 295645).

STROBE [27] and LEVEL recommendations [28] were followed.

### Setting and selection of participants

Eligible participants were EMS clinicians (i.e. paramedics) and non-registered professionals (e.g. Emergency Medical Technicians [EMTs]) delivering face-to-face patient care, employed by an NHS ambulance trust in the United Kingdom.

An opportunistic sample was recruited via social media and organisations’ internal communications. Informed consent was obtained in the baseline survey after providing study information. Access to the baseline survey was via an anonymous link, with individual diary study links issued to participants who provided their email address in their survey response. Participants completing all study elements were enrolled in a prize draw for three £50 vouchers to aid recruitment and reduce drop-out.

### Data collection

Data was collected using Qualtrics (Qualtrics, Provo, UT) (March-August 2022). The survey and diary study measures were developed for this study (Additional file 1). They were piloted with three EMS professionals and refined based on their feedback.

#### Baseline survey

The baseline survey covered demographics, feedback frequency and feedback environment. Demographic questions included professional role, years of EMS experience, sex, age and ethnicity. The feedback frequency questions were adapted from a large-scale US EMS feedback survey [15]. They included items such as ‘In the past 30 days, did you receive any feedback on the medical care you provided to a patient?’ scored on a dichotomous scale (‘yes/no’). If answered positively, it was followed by ‘How was this feedback provided? Verbal, by email, by text, written on paper, other’.

The feedback environment measure was based upon the shortened Feedback Environment Scale (FES) [29], which demonstrated excellent reliability for nurses (Cronbach’s alpha 0.90) [30]. The questions were adapted for the prehospital setting and reworded so as not to refer to a specific feedback source. Participants were asked to respond on a Likert-type scale ranging from 1 to 7 (strongly disagree-strongly agree) to statements such as ‘I receive useful feedback at work’ and ‘When I want feedback, this is readily available’. Once respondents provided ratings for each of the 14 items, the scores were aggregated. A high score on the FES generally indicates a positive perception of the feedback environment [29].

#### Diary entries

Immediately after completing the baseline survey, participants were sent a link to access their diary which remained open until the end of the data collection period. Participants were instructed to complete diary entries whenever they received feedback and were advised to log these entries as soon as possible to ensure accurate and timely recording. When logging a feedback event, participants were asked a series of multiple choice and structured response questions informed by Clinical Performance Feedback Intervention Theory [18], including, for example, ‘How quickly after the incident was the feedback provided?’ and ‘What effect do you think receiving this feedback had on your clinical practice/knowledge/confidence/sense of closure/job satisfaction/patient care/patient safety? Positive, negative or no effect’.

In this study, we differentiate between ‘negative feedback’ and ‘positive feedback’ based on the content and delivery of the feedback itself, i.e. the sign, nature or direction of feedback. ‘Negative feedback’ refers to feedback that highlights areas for improvement or points out errors, whereas ‘positive feedback’ focuses on reinforcing successful performance or praising achievements. Conversely, ‘feedback with a negative impact’ or ‘feedback with a positive impact’ refers to the subjective perception of the feedback’s effect on the recipient, as reported by the EMS professionals in their diaries. Thus, the same feedback can be perceived to have different impacts by different individuals.

### Data analysis

Quantitative analyses was undertaken in R (Version 4.1.3, R Core Team) [31] within RStudio [32] and qualitative analyses in NVivo (Version 12 Plus, QSR International). The detailed multilevel data analysis plan [33], study hypotheses and research models are described in Additional file 2.

Free-text qualitative responses in the baseline survey and diary entries were analysed using content analysis by early career paramedic researcher (CW) with input from the wider team of senior health services researchers (GJ, RL, JB). For the prevalence and predictors objectives, content analysis enabled the categorisation of free-text responses that participants has submitted under ‘other’ to either an existing category (e.g. ‘patient outcome feedback’) or development of a new category (e.g. ‘incident-reported feedback’). Within the hierarchical cluster analysis, qualitative insights enriched the interpretation of the quantitative results by providing contextual examples of perceived feedback impact among EMS professionals.

### Study size

Assuming 50/50 balanced binary predictors and normally distributed continuous predictors, 325 participants were required to detect any significant predictors of a medium-sized effect (i.e. Cohen’s d = 0.5) for prehospital feedback perceived as improving outcomes with 80% power, after adjustment for other variables [34]. The level-1 sample size was pre-specified by the research team as 10 diary entries was deemed an acceptable burden for each participant during stakeholder consultation. The power analysis was based on the basic research model, which included two level-2 predictors (role – binary, length in service – continuous) and two level-1 predictors (feedback content – categorical, solicited/unsolicited – binary).

### Statistical methods

Data on the individual-level variables (role, length in service, FES score) were collected during the baseline survey. Data on the diary-level independent variables (feedback content, feedback-seeking behaviour, formal/informal, source, sign, format, lag-time) and dependent variable (feedback outcome) were collected for each diary entry. FES scale reliability was examined using Cronbach’s alpha.

To describe feedback prevalence, descriptive statistics for baseline quantitative data were produced.

To identify predictors of receiving feedback in the last 30 days, baseline survey data were analysed using binary logistic regression (via ‘lme4’) [35]. Univariable logistic regression assessed individual associations between each predictor (e.g. role) and the outcome (i.e. having received feedback in the previous 30 days). Multivariable logistic regression included all predictors simultaneously that formed part of the simple or extended research model.

To identify predictors of perceived feedback efficacy, data generated via feedback-received diary entries were analysed using multilevel logistic regression with random intercepts to account for multiple recorded feedback instances per participant. The variables of interest were chosen based on Clinical Performance Feedback Intervention Theory [18] and qualitative exploratory studies of prehospital feedback [3, 7], for example feedback type, feedback-seeking behaviour and formal/informal. Continuous variables were grand-mean centred to improve the interpretation of the intercept values [36].

Akaike Information Criterion (AIC) [37] was used to compare models with the same outcome based on goodness-of-fit, whereby smaller AIC values indicate better fit. We did not adjust alpha for multiple comparisons due to deliberately favouring a higher Type I error rate relative to the potential for Type II error, as this was an exploratory study [38]. Analyses were conducted using complete cases, followed by sensitivity analyses dealing with missing data using the ‘mice’ R package [39].

To categorise perceived outcomes of receiving feedback, hierarchical cluster analysis was performed on the baseline data (using ‘ClustOfVar’ [40]). Cluster analysis is an exploratory analysis that identifies structures within the data and visualises them in a dendrogram (tree diagram) with outcomes that co-occur most frequently placed on branches closer together [41]. Clusters were labelled by the research team using thematic classification informed by previous research [3, 6].

## Results

### Characteristics of study participants

Two hundred and ninety-nine participants completed the baseline survey representing 13 of the 14 UK ambulance trusts (median 19, range 4–88 participants per trust). Of these, 105 completed 538 feedback-received diary entries (range 1–16, median 4).

Table 1 summarises participants’ baseline characteristics. Ethnicity was collapsed into a binary variable (white *n* = 290, minoritised ethnic group *n* = 8) to avoid identifying participants. Inferential statistics did not indicate that participants’ characteristics significantly differed between the baseline survey and diary entry stages. Comparison with national data for UK ambulance services [42] using chi-square tests at 0.05 significance level indicated that our study sample was representative in terms of ethnicity (*p* = 0.771), sex (*p* = 0.124) and age (*p* = 0.886).

The FES was found to have excellent internal consistency (alpha = 0.85 [95% CI 0.81 to 0.88]).

Of 299 baseline surveys, 78 (26.1%) were incomplete. Missing values varied from 0.3 to 25.1%.

**Table 1:** Characteristics of study participants

	Baseline survey	Diary entries
**Number of participants**, n	299	105
**Role**, n (%)		
EMT	59 (19.7)	16 (15.2)
Paramedic	239 (79.9)	89 (84.8)
**Age in years**, median (IQR)	36 (29.0–45.0)	38 (30.5–45.0)
**Sex**, n (%)		
Female	120 (40.1)	39 (37.1)
Male	177 (59.2)	66 (62.9)
Not stated	2 (0.7)	0 (0)
**Ethnicity**, n (%)		
Minoritised ethnic group	8 (2.7)	2 (1.9)
White	290 (97.0)	103 (98.1)
Not stated	1 (0.3)	0 (0)
**Years of work experience**, median (IQR)	7 (3.7–13.4)	9 (4.5–14.4)
**FES score**, mean ± SD	53.63 ± 14.22	52.72 ± 13.09
**Presence of formal feedback initiative**, n (%)		
Yes	68 (22.7)	26 (24.8)
No	231 (77.3)	79 (75.2)

### Feedback prevalence and types

Table 2 describes the characteristics of feedback prevalence from the baseline data and diary entries.

Of the 299 participants completing the baseline survey, 215 (71.9%) indicated that they had received feedback in the last 30 days, with patient outcome feedback being the most frequently received (*n* = 149, 42.8%). Feedback was predominantly provided in verbal format (*n* = 157, 73.0%) and was informal (*n* = 189, 80.4%).

**Table 2:** Characteristics of feedback prevalence at baseline and during diary study

	Baseline feedback received (*N* = 215), n(%)	Diary entries feedback received (*N* = 538), n(%)
**Type**		
Patient outcome feedback	149 (42.8)	226 (42.0)
Patient experience feedback	88 (25.3)	108 (20.1)
Clinical performance feedback	111 (31.9)	201 (37.4)
Incident-prompted feedback	-	2 (0.4)
Post-event debriefing	-	1 (0.2)
**Source** *(multiple selections possible)*		
Non-ambulance healthcare professionals	114 (33.9)	202 (37.5)
EMS professionals or managers	132 (39.3)	180 (33.5)
Patients/relatives	85 (25.3)	147 (27.3)
Other	5 (1.5)	9 (1.7)
**Format**		
Electronic	41 (19.1)	90 (16.7)
Verbal	157 (73.0)	414 (77.0)
Written	16 (7.4)	30 (5.6)
Other	1 (0.5)	4 (0.7)
**Lag time**		
Immediate or within 1 day	130 (45.8)	370 (68.8)
2–3 days	53 (18.7)	45 (8.4)
4–7 days	27 (9.5)	41 (7.6)
8–14 days	28 (9.9)	24 (4.5)
More than 14 days	46 (16.2)	58 (10.8)
**Feedback-seeking behaviour**		
Unsolicited	143 (53.6)	335 (62.3)
Solicited	124 (46.4)	203 (37.7)
**Formal/informal**		
Formal	46 (19.6)	87 (16.2)
Informal	189 (80.4)	451 (83.8)
**Sign** (i.e. nature or direction of feedback)		
Positive	Not collected	383 (71.6)
Neutral	Not collected	91 (17.0)
Negative	Not collected	16 (3.0)
Mixed	Not collected	45 (8.4)

### Predicted likelihood of receiving feedback

**Fig. 1:**
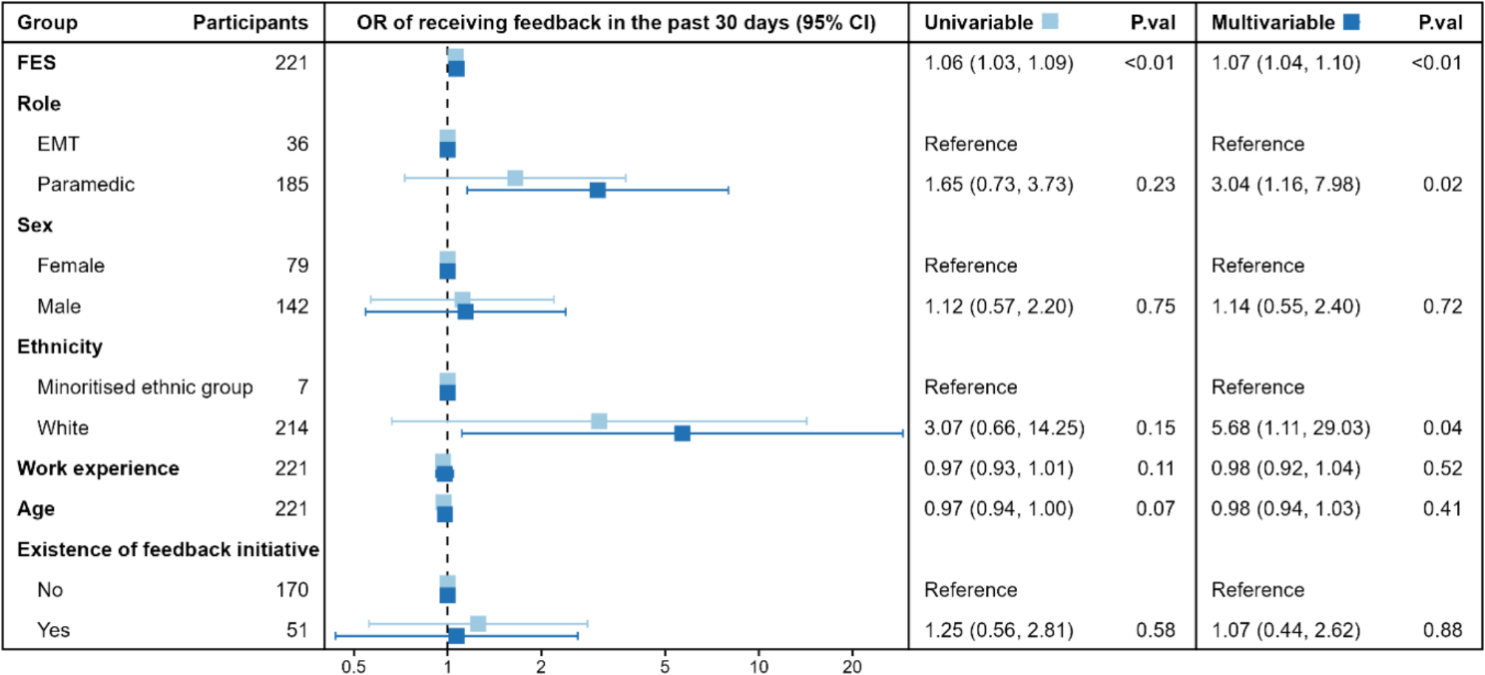
Forest plot of factors associated with receiving feedback in the past 30 days

The likelihood of receiving feedback in the past 30 days was higher for those with a supportive feedback environment (aOR 1.07 [1.04, 1.10]), meaning that each one-point increase in FES increased the odds of receiving feedback by 7% (see Fig. 1). Participants in paramedic roles had three times the estimated odds of receiving feedback than EMTs (aOR 3.04 [1.14, 8.00]). Those of white ethnicity had five times the estimated odds of receiving feedback compared with minoritised ethnic group participants (aOR 5.68 [1.01, 29.73]); although, the wide confidence interval indicates a high level of uncertainty in this estimate. The sensitivity analysis (Additional file 3) indicated that when missing data was imputed, ethnicity did not predict the likelihood of receiving feedback (aOR 3.34 [0.71, 15.71]).

### Perceived outcomes of feedback

Feedback outcomes were categorised into three clusters following a visual inspection of the dendrogram from the hierarchical cluster analysis and stability of the partitions (Additional file 4). Cluster 1 (‘*professional development*’) encompassed clinical practice and knowledge, Cluster 2 (‘*personal wellbeing’*) encompassed closure, confidence and job satisfaction, and Cluster 3 (‘*service outcomes’*) encompassed patient care and patient safety.

**Fig. 2:**
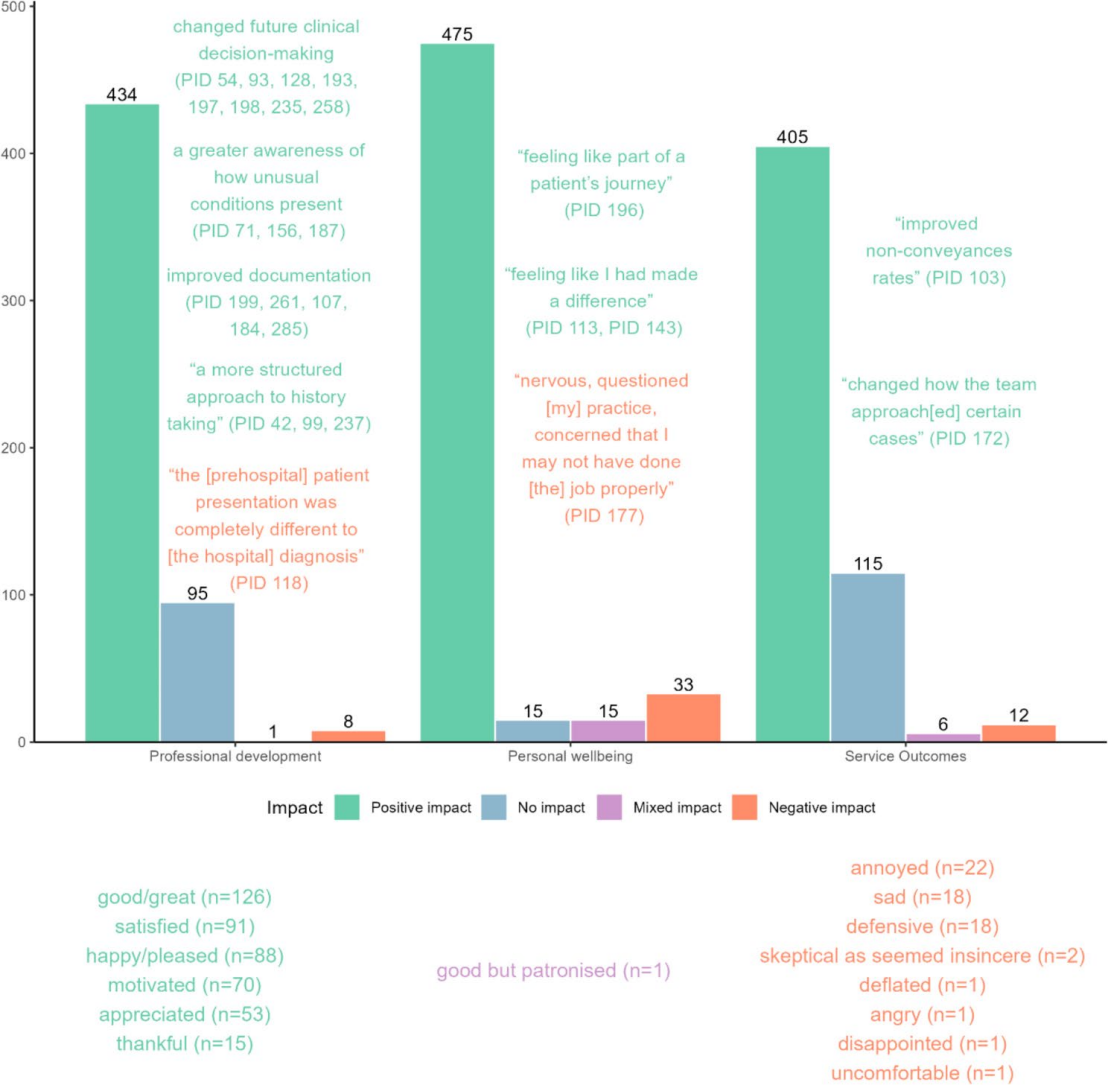
Perceived impact within each outcome cluster

Figure 2 describes the count of perceived positive, negative, mixed and no impact within each feedback outcome cluster and contextual examples from qualitative findings. Overall, feedback was perceived to have a positive impact. The 33 feedback events resulting in negative affective responses were reported by 25 participants, who had lower FES scores and received punitive feedback that was predominantly negative, unsolicited and provided by EMS professionals.

### Predicted likelihood of feedback efficacy

Additional file 5 summarises the results of the univariable and multivariable multilevel analyses identifying predictors of feedback efficacy. Sensitivity analyses (Additional file 6) indicated that missing data had some effect in the univariable analyses but little effect in the multivariable multilevel analyses. The intraclass correlation coefficient (ICC [ICC_Professional_=0.25, ICC_Personal_=0.19, ICC_Service_=0.24]) indicated that a moderate amount of the variablity in feedback having a positive impact was explained at a participant level, rather than at the level of individual feedback events.

Comparing the AICs for the basic and extended research model suggested that the extended research model was the best fit for all three outcome clusters. The extended research model indicated that feedback-seeking behaviour and FES were statistically significant predictors of feedback efficacy. Solicited feedback was more likely to improve professional development (aOR 3.35 [1.68, 6.69]) and personal wellbeing (aOR 2.58 [1.19, 5.56]) than unsolicited feedback. A one-point increase in FES led to a predicted 4% increase in the odds of feedback positively affecting personal wellbeing (aOR 1.04 [1.01, 1.07]) and a 3% increase for service outcomes (aOR 1.03 [1.00, 1.06]).

## Discussion

In total, 215 (71.9%) participants indicated that they had received feedback in the last 30 days with patient outcome feedback most received (*n* = 149, 42.8%). Significant predictors for receiving feedback were a paramedic role and a workplace with a positive feedback-seeking culture. Participants reported that feedback affected personal wellbeing (closure, confidence, job satisfaction), professional development (clinical practice, knowledge) and service outcomes (patient care, patient safety). Solicited feedback was more likely to positively affect personal and professional development than unsolicited feedback.

Compared to US studies, our participants reported a slightly higher prevalence of receiving feedback in the past 30 days: 71.9% compared to 50.0% [16] and 69.4% [15]. This could be because our study provided clearer specification of feedback through definitions provided to participants.

Consistent with other studies, feedback was mostly received in verbal format (73.0%) and provided by a mixture of EMS professionals (39.3%), non-ambulance healthcare professionals (33.9%) and patients or relatives (25.3%) [15, 16]. Patient outcome feedback was the type most frequently received by our participants (42.8%), which differed from the largest US study on this topic in which receipt of clinical performance feedback dominated [15].

The limited reporting of debriefing in our study was surprising given that recent research identified debriefing as a prehospital feedback type. Post-event debriefing is designed to help staff process and learn from unusual or critical events [43]. Although some ambulance services have implemented debriefing programs to support staff [44], these sessions – which focus on understanding and making sense of events [45] – were less commonly reported in our study. This discrepancy may be explained by the rarity of critical incidents requiring post-event debriefing and the perception that debriefing is distinct from routine feedback on clinical performance or patient outcomes.

In contrast to previous studies of prehospital feedback [14–16], years of experience were not a significant predictor of receiving feedback in our study. However, we did identify several novel predictors of receiving feedback, such as paramedic role and a workplace with a supportive feedback culture as indicated by high FES scores. Paramedics may receive more feedback compared with EMTs because they take the lead on more acute cases and are therefore in a better position to actively seek feedback, as indicated by 38.6% (*n* = 180) of feedback for paramedics being solicited compared with only 31.9% (*n* = 23) for EMTs. It may also be that paramedics have become used to receiving enhanced feedback during undergraduate training or the newly qualified paramedic period and are therefore continuing to seek enhanced feedback provision [3]. The broader feedback literature offers theoretical support regarding feedback exchanges being affected by social categories such as race, gender, age and sexual orientation, in that staff with minority characteristics are less likely to actively seek feedback [46]. Further understanding how personal characteristics influence EMS feedback interactions is vital to promote equity and inclusion within feedback theory and practice.

Our analysis indicates that solicited feedback was more likely to improve professional development and personal wellbeing than unsolicited feedback. This may be due to solicited feedback being timelier, more relevant and originating from a more credible source as the recipient has some control over whom they approach, compared with unsolicited feedback. Overall this probably reflects the limitations of the existing prehospital feedback provision in regards to timeliness, relevance and credibility, rather than solicited feedback being an ultimate desirable goal [7].

The positive effects of prehospital feedback on quality of care and professional development were synthesised in a recent systematic review [6], but EMS professionals in our study also perceived that feedback positively affects personal outcomes such as closure (68.8%), confidence (83.1%) and job satisfaction (81.8%). This confirms suggestions from qualitative and survey studies that feedback for EMS professionals can support staff wellbeing and job satisfaction [3, 4, 7, 16].

Our study also highlights the importance of feedback delivery, demonstrating that the perceived negative impacts of feedback are influenced not only by its content (e.g. a negative patient outcome), but also by how it is delivered (“*made me feel uncomfortable*”) and the credibility of the feedback source (“*not genuine*”). In the broader audit and feedback literature, credibility of the feedback source is known to influence feedback effectiveness [5, 47]. Brehaut et al. [47] emphasize that credible feedback is less likely to provoke defensive reactions and more likely to be effective. Additionally, a strong relationship between the feedback provider and recipient encourages feedback-seeking behaviour [48]. Thus, the manner of delivery and the provider’s credibility are crucial for minimising negative emotional responses and improving feedback outcomes.

### Implications for research and practice

Further research should include developing theory-informed measures to evaluate how prehospital feedback initiatives impact professional practice, personal wellbeing and service outcomes. Observational studies within EMS should be conducted to deepen our understanding of solicited and unsolicited feedback, the delivery of negative feedback and the influence of personal characteristics on EMS feedback interactions and engagement. A particular area in need of further research are minoritised ethnic EMS professionals. Further research should also focus on what feedback EMS professionals want to receive.

Change in clinical practice should focus on designing and robustly evaluating feedback provision for EMS professionals. All EMS professionals should be enabled to make better use of the feedback they have access to. EMTs should be supported to actively seek feedback to address the current feedback inequity, which places them at a disadvantage when it comes to development of professional competency and performance. Care should be taken in feeding back service level outcomes to frontline EMS professionals to ensure that the feedback is relevant and actionable at their level.

Tailoring feedback interventions to support personal wellbeing is most likely to be perceived by EMS professionals to have positive impacts than those targeting professional development or service outcomes. The benefits of feedback for staff wellbeing should be formally recognised by ambulance services given the potential to mitigate workforce challenges, such as burnout, retention and recruitment. Feedback targeting personal wellbeing may also do harm and organisations should adequately support EMS professionals when receiving feedback.

### Strengths and limitations

This was the first study to assess feedback prevalence within the UK EMS population and to explore the associated contextual factors and outcomes. This study was limited by the high drop-out rate (*n* = 299 participants at baseline, *n* = 105 logging diary entries), though this is typical of diary studies generally [49]. A further limitation is that while participants were instructed to log diary entries whenever they received feedback, delays in entry completion likely led to omissions and contributed to lower participation rates. To combat high dropout in future diary studies, researchers could offer greater incentives or further reduce survey length. However, using diary methods was a novel way to assess feedback prevalence that reduced recall bias and provided reliable within-person data. Testing for differences between the prospective diary entries and retrospective baseline data to quantify recall bias indicated significantly shorter lag times (*p* < 0.001) and a higher proportion of unsolicited feedback (*p* = 0.018) for the prospectively collected data, suggesting that retrospective data collection may not be reliable for feedback in EMS.

Despite data collection taking place during the early post-pandemic period when the backlog of health needs were emerging, the large number of NHS staff that participated and feedback events that were reported, indicate an appetite for feedback research from EMS professionals. However, this study was unable to recruit to target. Challenges related to the demanding schedules and limited availability for research participation of the target NHS staff group, combined with reliance on voluntary participation, are likely to have contributed to the relatively low response rate. Future research should explore alternative recruitment strategies to enhance participation rates within this professional context.

Comparison with national data for UK ambulance services [42] indicated that our study sample was representative of UK EMS but it remains unclear to what extent these findings might be replicated in the health systems of other countries. We acknowledge that collapsing our ethnicity variable into binary categories limits our conclusions regarding specific minoritised ethnic groups. The divergence between the complete case analysis and the multiple imputation sensitivity analysis regarding whether ethnicity predicted the likelihood of receiving feedback suggests this predictor may not be very robust. However, as feedback is mostly positive, this is a potential inequality and needs further investigation. Future studies should specifically target minority group participation, particularly as the literature suggests that social identity and race influence feedback-seeking behaviour [46].

Another limitation is the absence of triangulation of sources. Feedback is a two-way process [15, 50], and relying solely on self-reported data from EMS professionals may not fully capture its dynamics. Including perspectives from feedback providers could have provided a more comprehensive understanding of the feedback process. Future research should incorporate multiple sources to enhance the depth and accuracy of findings.

## Conclusions

In conclusion, our study provides valuable insights into the prevalence, predictors and outcomes of feedback provision within the UK EMS context. Our findings underscore the importance of feedback in enhancing not only clinical practice and service outcomes but also personal wellbeing and job satisfaction among EMS professionals. However, the delivery of feedback emerged as a critical factor influencing its effectiveness, highlighting the need for attention to credibility and sensitivity in feedback delivery. Addressing feedback inequities, particularly among non-registered EMS professionals and minoritised groups, is crucial for promoting workforce development and ensuring equitable access to development opportunities. Overall, this study suggests that EMS workplaces need to develop a culture that encourages feedback-seeking by ensuring high-quality positive and negative feedback is readily available and provided by a credible source to strengthen the impact of feedback for EMS professionals on clinical decision-making and staff wellbeing.

## Electronic supplementary material

Below is the link to the electronic supplementary material.

Supplementary Material 1: Survey and diary study measures

Supplementary Material 2: In-depth data analysis plan, study hypotheses and theoretical models

Supplementary Material 3: Sensitivity analysis for the predicted likelihood of receiving feedback

Supplementary Material 4: Clustering dendogram and stability of the partitions

Supplementary Material 5: Results of the univariable and multivariable analyses (including basic and extended research models)

Supplementary Material 6: Sensitivity analyses of the predicted likelihood of feedback efficacy

## Data Availability

The datasets generated and analysed during the current study are not publically available as sharing the raw data would violate the agreement to which participants consented; however, the datasets are available from the corresponding author on reasonable request.

## Abbreviations

AIC: Akaike Information Criterion
ED: Emergency Department
EMS: Emergency Medical Services
EMT: Emergency Medical Technician
FES: Feedback Environment Scale
NHS: National Health Service
OR: Odds Ratio
