# Supplementary material for: Prevalence, predictors and outcomes of self-reported feedback for EMS professionals: a mixed-methods diary study
Source: BMC Emerg Med. 2024 Sep 13;24:165. doi: 10.1186/s12873-024-01082-y (PMC11395609; doi:10.1186/s12873-024-01082-y)
Supplement: Supplementary file 1 — Supplementary Material 1: Survey and diary study measures [file 12873_2024_1082_MOESM1_ESM.docx]

**Additional file 1: Survey and diary study measures**

**Baseline**

**Background details**

- Please select your employing NHS Ambulance Service Trust: *List of 13 UK NHS Ambulance Trusts*
- What is your professional role?
  - Paramedic registered with HCPC (including all paramedic roles for example specialist paramedic, advanced paramedic, critical care paramedic, senior paramedic team leader etc.), Non-registered emergency ambulance staff (e.g. Emergency Medical Technician, Emergency Care Assistant etc.)
- In total, how long have you worked in the ambulance service (in years and months): __ Years _ Months
- What is your sex? Male, female, Other (please specify): ______, Prefer not to say
- What is your age? __ Years
- What is your ethnicity? White, Mixed or Multiple ethnic groups, Asian or Asian British, Black/African/Caribbean or Black British, Other ethnic group, Prefer not to say

**Experience of feedback**

- In the past 30 days, did you receive any feedback about the following? *Please tick all that apply.*
  - Your performance at work (e.g. the medical care you provided to a patient, response times, on-scene times, non-conveyance rates), The outcome of a patient after you attended to them, Patient satisfaction, Your documentation associated with attending to a patient, Other (*please specify*): ____________
- Who provided you with feedback? *Please tick all that apply.*
  - Your crew mate or colleague, Your immediate line manager, Senior ambulance manager(s), Staff at the receiving facility, Patients/relatives, Other (*please specify*): ____________
- How did you receive feedback? *Please tick all that apply.*
  - Verbal, By email, By text, Written on paper, Other (*please specify*): ___
- How soon after the incident was the feedback provided? *Please tick all that apply.*
  - Within 1 day of the incident, 2-3 days after the incident, 4-7 days after the incident, 8-14 days after the incident, More than 14 days after the incident
- When you received feedback, was this sought by you or provided without seeking? *Please tick all that apply.*
  - Sought, Provided without seeking
- In the past 30 days, has any of the feedback that you’ve received, resulted in a change to professional practice at an individual, team or organisational level? If yes, please describe it here:
  - Yes: Individual: ___, Team: ___, Organisational: ___; No
- When you received feedback, was this through formal (e.g. paramedic post-box scheme, official debrief, performance appraisal) or informal channels? *Please tick all that apply.*
  - Formal, Informal
- In your local area, is there an initiative that provides feedback to emergency ambulance staff?
  - Yes: Please describe it briefly here: ___, What do you think about this initiative?___; No
- Is there an area of your clinical practice that you would like to receive more feedback on than you currently do? Please describe it here, again remembering not to include confidential information about yourself, patients or colleagues: ___

**Feedback environment**

Please indicate your answer on a scale from 1 (strongly disagree) to 7 (strongly agree):

|  | **Source Credibility** | Strongly Disagree → Strongly Agree | | | | | | |
| --- | --- | --- | --- | --- | --- | --- | --- | --- |
| 1.a. | The person providing me with feedback is generally familiar with my clinical practice. | 1 | 2 | 3 | 4 | 5 | 6 | 7 |
| 1.b. | I think the person providing me with feedback is fair when evaluating my clinical practice. | 1 | 2 | 3 | 4 | 5 | 6 | 7 |
|  | **Feedback Quality** | Strongly Disagree → Strongly Agree | | | | | | |
| 2.a. | I receive useful feedback at work. | 1 | 2 | 3 | 4 | 5 | 6 | 7 |
| 2.b. | The feedback I receive helps me to do my job. | 1 | 2 | 3 | 4 | 5 | 6 | 7 |
|  | **Feedback Delivery** | Strongly Disagree → Strongly Agree | | | | | | |
| 3.a. | When I access or receive feedback, the person providing me with feedback is considerate of my feelings. | 1 | 2 | 3 | 4 | 5 | 6 | 7 |
| 3.b. | When I access or receive feedback, this is provided in a sensitive way. | 1 | 2 | 3 | 4 | 5 | 6 | 7 |
|  | **Favourable Feedback** | Strongly Disagree → Strongly Agree | | | | | | |
| 4.a. | I receive feedback that tells me when I do a good job at work. | 1 | 2 | 3 | 4 | 5 | 6 | 7 |
| 4.b. | I frequently receive positive feedback at work. | 1 | 2 | 3 | 4 | 5 | 6 | 7 |
|  | **Unfavourable Feedback** | Strongly Disagree → Strongly Agree | | | | | | |
| 5.a. | On those occasions when my job performance falls below what is expected, the person providing me with feedback lets me know. | 1 | 2 | 3 | 4 | 5 | 6 | 7 |
| 5.b. | On those occasions when I make a mistake at work, the person providing me with feedback tells me. | 1 | 2 | 3 | 4 | 5 | 6 | 7 |
|  | **Feedback Availability** | Strongly Disagree → Strongly Agree | | | | | | |
| 6.a. | When I want feedback, this is readily available. | 1 | 2 | 3 | 4 | 5 | 6 | 7 |
| 6.b. | The person providing me with feedback is too busy to give me feedback. | 1 | 2 | 3 | 4 | 5 | 6 | 7 |
|  | **Promotes Feedback Seeking** | Strongly Disagree → Strongly Agree | | | | | | |
| 7.a. | I feel comfortable asking for feedback at work. | 1 | 2 | 3 | 4 | 5 | 6 | 7 |
| 7.b. | The person providing me with feedback encourages me to ask for feedback whenever I am uncertain. | 1 | 2 | 3 | 4 | 5 | 6 | 7 |

**Feedback received**

Please indicate in the following, which characteristics apply to the feedback that you have just received.

- Content of the feedback:
  - Your performance at work (e.g. the medical care you provided to a patient, response times, on-scene times, non-conveyance rates), The outcome of a patient after you attended to them, Patient satisfaction, Your documentation associated with attending to a patient, Other? (*please specify*): ____________
- Which of the following categories does this incident fit into? (*select all that apply*)
  - Non-conveyance, On-scene times or call-to-hospital times, Major trauma, Paediatrics, Cardiac arrest, Other cardiac condition (e.g. myocardial infarction), Respiratory condition (e.g. asthma), Neurological condition (e.g. stroke), Gastrointestinal condition (e.g. acute abdomen), Other? (*please specify*): ____________
- Source of the feedback:
  - Your crew mate or prehospital colleague, Your immediate line manager, Senior ambulance managers, Staff at the receiving facility, Patients/relatives, Other (*please specify*): ____________
- Overall message of the feedback:
  - Positive, Negative, Mixed, Neutral
- Format that the feedback was provided in:
  - Verbal, By email, By text, Written on paper, Other (*please specify*): ____________
- How quickly after the incident was the feedback provided?: Within _______ days
- Did you seek out the feedback or was it provided to you without seeking?
  - Sought, Provided without seeking
- Was this feedback provided to you through formal (e.g. paramedic post-box scheme, official debrief, performance appraisal) or informal channels?
  - Formal, Informal
- How did receiving this feedback make you feel? _____________________
- Did the feedback answer your question?
  - Yes, No, Not applicable
- After receiving the feedback, have you engaged (or do you plan to engage) in any of the following learning activities? *Tick all that apply.*
  - Read professional books/journals, Asked my supervisor or colleagues for advice, Took part in training opportunities, Thought about if the feedback regarding my work matches my own judgement, Reflected on what exactly I did right/wrong, Changed my clinical practice, Observed how others work, Searched for help/solutions on the internet, Discussed the feedback with my line manager/colleagues/others, Passed on my knowledge/skills to others, Other *(please specify*): _______________
- What effect do you think receiving this feedback has had on…….? *Please check positive, negative or no effect on each line.*
  - Your clinical practice, Your knowledge, Your confidence, Your sense of closure, Your job satisfaction, Patient care, Patient safety, Other (*please specify*): ___________
- Lastly, on a scale of 1-7 how would you rate the usefulness of the feedback you received? *(1 – not useful at all, 7 – extremely useful)*
