# Supplementary material for: Prevalence, predictors and outcomes of self-reported feedback for EMS professionals: a mixed-methods diary study
Source: BMC Emerg Med. 2024 Sep 13;24:165. doi: 10.1186/s12873-024-01082-y (PMC11395609; doi:10.1186/s12873-024-01082-y)
Supplement: Supplementary file 2 — Supplementary Material 2: In-depth data analysis plan, study hypotheses and theoretical models [file 12873_2024_1082_MOESM2_ESM.docx]

**Additional file 2: In-depth data analysis plan, study hypotheses and theoretical models**

For the multilevel modelling used to examine feedback received diary entries, we followed the three-step procedure developed by Sommet and Morselli (41): *Step 1* – ran an empty model and calculated the intra-class correlation coefficient (ICC) to quantify the extent to which the variability of feedback effects was due to clustering of diary entries within participants, *Step 2* – ran an intermediate model to determine whether allowing the effects of the diary-level variables to vary from one participant to another (‘random slope variance’) improved the model fit, *Step 3* – ran a final model and interpreted the Odds Ratio (OR) and 95% Confidence Interval (CIs) to determine whether the data support our hypotheses and theoretical models outlined below. The three steps were conducted for the basic research model (i.e. role, length in service, feedback type, feedback-seeking behaviour) and the extended research model (adding FES, formal/informal). This was repeated for the three feedback outcomes (i.e. personal development, professional development and service outcomes), resulting in six final multivariable models.

| **#** | **Hypothesis** | **Method of analysis** | **Number of data items** | **Valid n** |
| --- | --- | --- | --- | --- |
| 1 | Recipient and contextual characteristics will influence the likelihood of having received feedback in the past 30 days | Univariable + multivariable binomial logistic regression | 8 | 221 |
| 2 | Feedback content (L_1_) will influence the feedback outcome (L_1_) | Multilevel univariable binomial logistic regression | 2 | 535 |
| 3 | Feedback source (L_1_) will influence the feedback outcome (L_1_)* |  | 2 | 535 |
| 4 | Feedback sign (L_1_) will influence the feedback outcome (L_1_)* |  | 2 | 535 |
| 5 | Feedback format (L_1_) will influence the feedback outcome (L_1_)* |  | 2 | 535 |
| 6 | Lag-time (L_1_) will influence the feedback outcome (L_1_)* |  | 2 | 535 |
| 7 | Feedback-seeking behaviour (L_1_) will influence the feedback outcome (L_1_) |  | 2 | 535 |
| 8 | Formal/informal (L_1_) will influence the feedback outcome (L_1_) |  | 2 | 535 |
| 9 | Feedback environment scale (L_2_) will influence the feedback outcome (L_1_) |  | 2 | 535 |
| 10 | Role (L_2_) will influence the feedback outcome (L_1_) |  | 2 | 535 |
| 11 | Sex (L_2_) will influence the feedback outcome (L_1_)* |  | 2 | 535 |
| 12 | Ethnicity (L_2_) will influence the feedback outcome (L_1_)* |  | 2 | 535 |
| 13 | Years of work (L_2_) experience will influence the feedback outcome (L_1_) |  | 2 | 535 |
| 14 | Age (L_2_) will influence the feedback outcome (L_1_)* |  | 2 | 535 |
| 15 | Feedback content (L_1_), feedback-seeking behaviour (L_1_), person role (L_2_) and length in service (L_2_) will influence if there is a perceived positive change to professional development following the feedback incident (L_1_) | Multilevel multivariable binomial logistic regression | 5 | 535 |
| 16 | Feedback content (L_1_), feedback-seeking behaviour (L_1_), person role (L_2_) and length in service (L_2_) will influence if there is a perceived positive change in personal wellbeing following the feedback incident (L_1_) |  | 5 | 535 |
| 17 | Feedback content (L_1_), feedback-seeking behaviour (L_1_), person role (L_2_) and length in service (L_2_) will influence if there is a perceived positive change in service outcomes following the feedback incident (L_1_) |  | 5 | 535 |
| 18 | Feedback content (L_1_), feedback-seeking behaviour (L_1_), formal/informal (L_1_), FES (L_2_), person role (L_2_) and length in service (L_2_) will influence if there is a perceived positive change to professional development following the feedback incident (L_1_) |  | 7 | 535 |
| 19 | Feedback content (L_1_), feedback-seeking behaviour (L_1_), formal/informal (L_1_), FES (L_2_), person role (L_2_) and length in service (L_2_) will influence if there is a perceived positive change in personal wellbeing following the feedback incident (L_1_) |  | 7 | 535 |
| 20 | Feedback content (L_1_), feedback-seeking behaviour (L_1_), formal/informal (L_1_), FES (L_2_), person role (L_2_) and length in service (L_2_) will influence if there is a perceived positive change in service outcomes following the feedback incident (L_1_) |  | 7 | 535 |

* indicates hypotheses that were not identified a priori

**Basic research model**

**
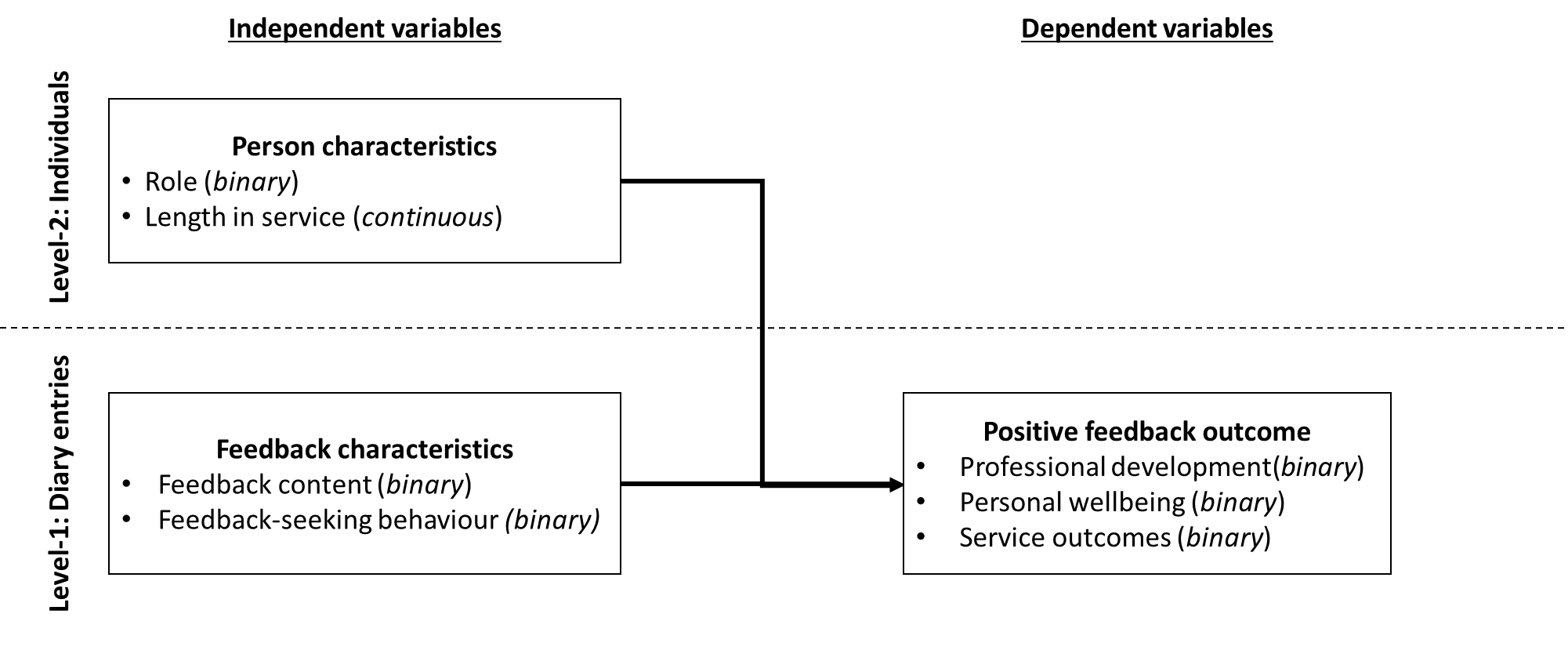
**

**Extended research model**

**
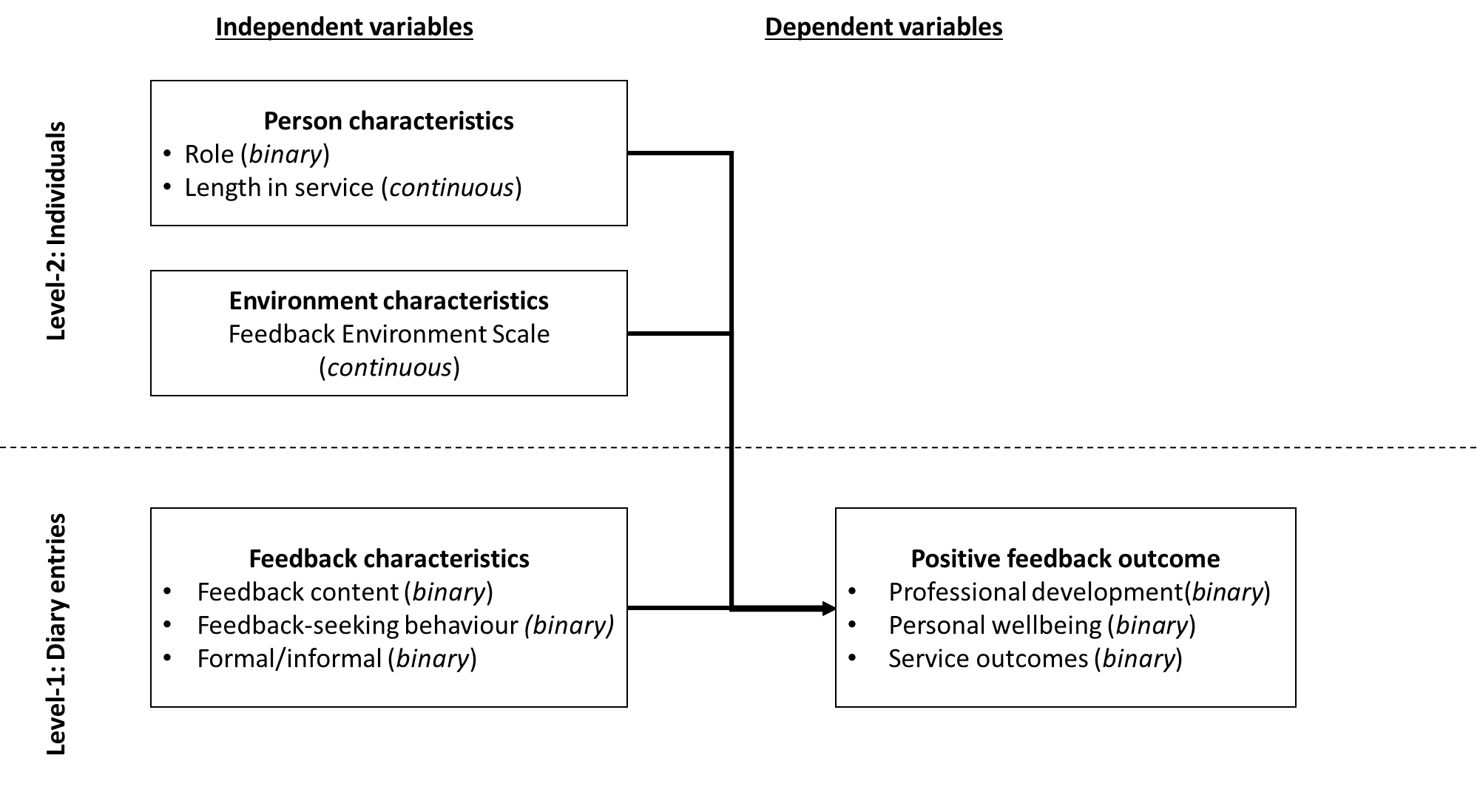
**
