# Supplementary material for: Prevalence, predictors and outcomes of self-reported feedback for EMS professionals: a mixed-methods diary study
Source: BMC Emerg Med. 2024 Sep 13;24:165. doi: 10.1186/s12873-024-01082-y (PMC11395609; doi:10.1186/s12873-024-01082-y)
Supplement: Supplementary file 3 — Supplementary Material 3: Sensitivity analysis for the predicted likelihood of receiving feedback [file 12873_2024_1082_MOESM3_ESM.docx]

**Additional file 3: Sensitivity analyses for the predicted likelihood of receiving feedback**

|  | **Univariable**  **OR (95% CI), p-value** | **Multivariable**  **aOR (95% CI), p-value** |
| --- | --- | --- |
| **FES** *(continuous)* | 1.06 (1.03, 1.09)*, p<0.001* | 1.06 (1.03, 1.09), p<0.001* |
| **Role** *(binary)* (ref=EMT) | | |
| Paramedic | 2.02 (1.11, 3.68), p=0.021* | 3.24 (1.58, 6.66), p=0.001* |
| **Sex** *(binary)* (ref=Female) | | |
| Male | 1.11 (0.73, 2.04), p=0.457 | 1.21 (0.68, 2.16), p=0.508 |
| **Ethnicity** *(binary)* (ref=Non-white) | | |
| White | 1.60 (0.37, 6.92), p=0.526 | 3.34 (0.71, 15.71), p=0.126 |
| **Years of work experience** *(continuous)* | 0.97 (0.94, 1.00), p=0.038 | 0.97 (0.92, 1.02), p=0.173 |
| **Age** *(continuous)* | 0.98 (0.95, 1.00), p=0.050 | 0.99 (0.96, 1.03), p=0.683 |
| **Existence of feedback initiative** *(binary)* (ref=no) | | |
| Yes | 1.88 (0.96, 3.66), p=0.065 | 1.68 (0.79, 3.55), p=0.177 |
