# Supplementary figures and images for: Prevalence, predictors and outcomes of self-reported feedback for EMS professionals: a mixed-methods diary study
Source: BMC Emerg Med. 2024 Sep 13;24:165. doi: 10.1186/s12873-024-01082-y (PMC11395609; doi:10.1186/s12873-024-01082-y)

Cluster Dendrogram

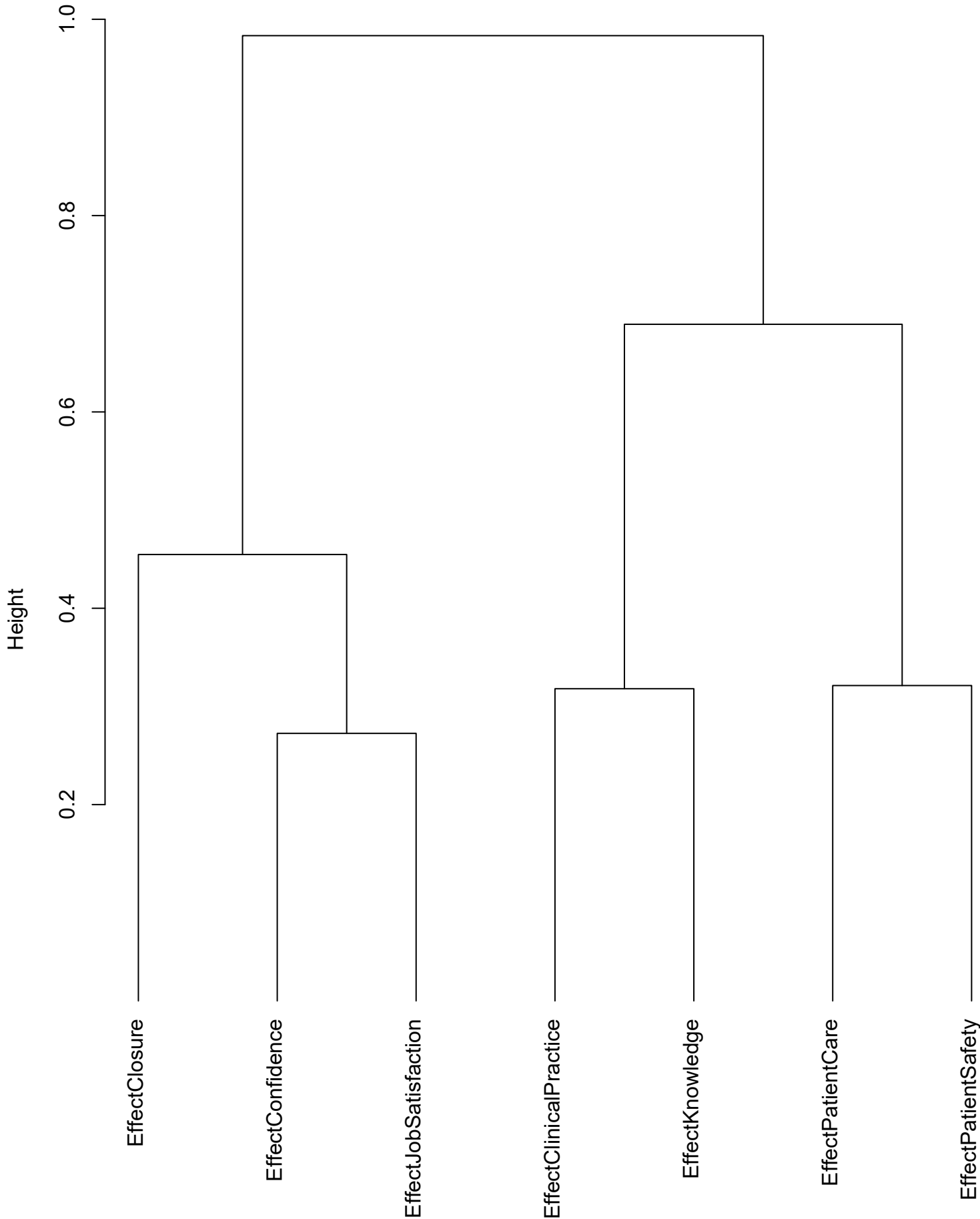

**Stability of the partitions**

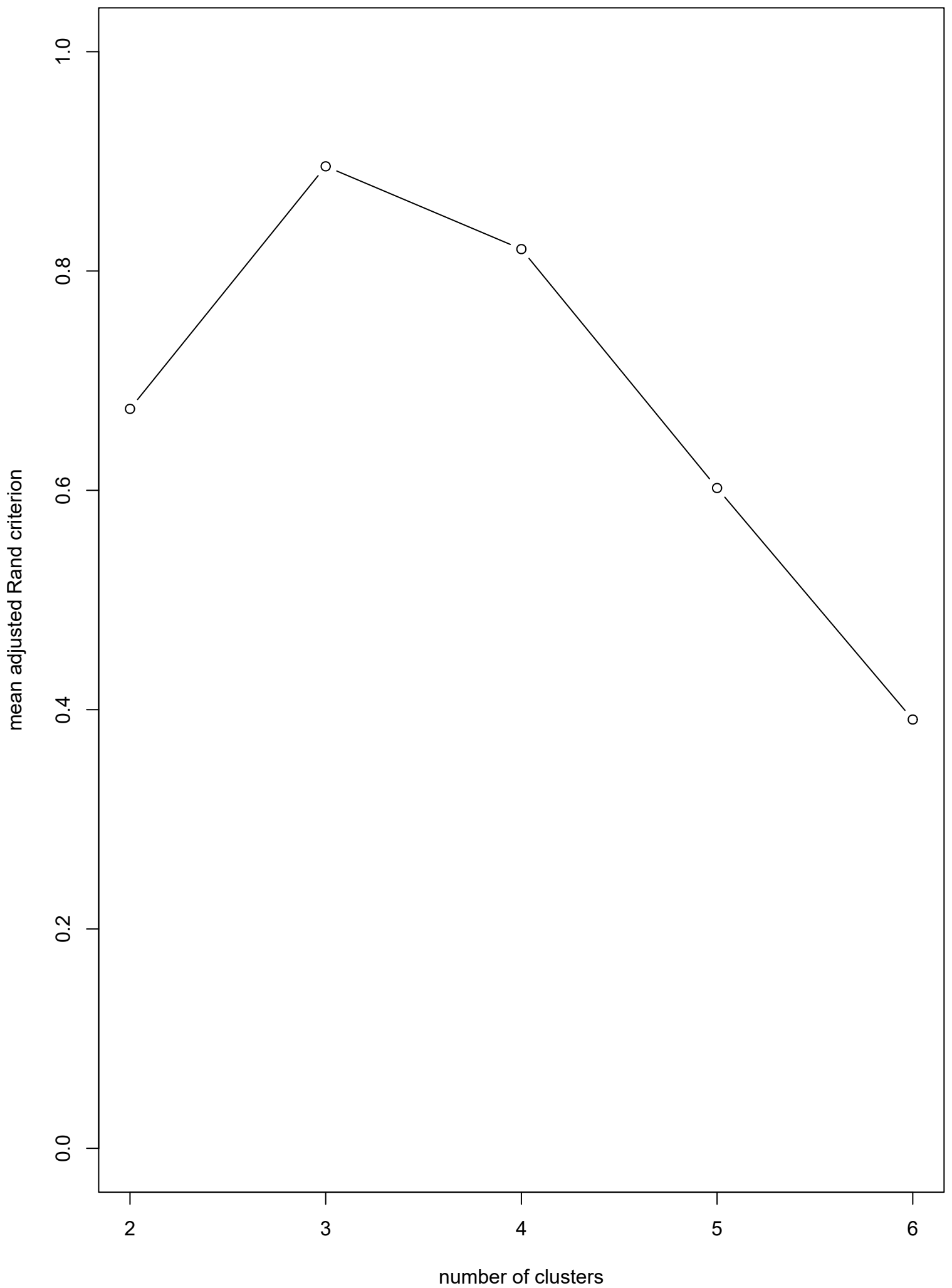

Supplement: Supplementary file 4 — Supplementary Material 4: Clustering dendogram and stability of the partitions [file 12873_2024_1082_MOESM4_ESM.pdf]
