# Supplementary material for: Prevalence, predictors and outcomes of self-reported feedback for EMS professionals: a mixed-methods diary study
Source: BMC Emerg Med. 2024 Sep 13;24:165. doi: 10.1186/s12873-024-01082-y (PMC11395609; doi:10.1186/s12873-024-01082-y)
Supplement: Supplementary file 5 — Supplementary Material 5: Results of the univariable and multivariable analyses (including basic and extended research models) [file 12873_2024_1082_MOESM5_ESM.docx]

**Additional file 5: Results of the univariable and multivariable analysis (including basic and extended research model)**

|  | **Professional development** | | |
| --- | --- | --- | --- |
|  | **Univariable analysis**  **OR (95% CI), p-value** | **Multivariable - Basic research model**  **aOR (95% CI), p-value** | **Multivariable – Extended research model**  **aOR (95% CI), p-value** |
| **Content** (*categorical, diary-level variable*) (ref = Patient outcome feedback) | | | |
| Patient experience | 0.38 (0.20, 0.75), p=0.005* | 0.60 (0.29, 1.24), p=0.166 | 0.64 (0.29, 1.40), p=0.266 |
| Clinical performance | 0.70 (0.39, 1.26), p=0.235 | 0.96 (0.51, 1.79), p=0.889 | 0.92 (0.48, 1.79), p=0.811 |
| **Source** *(categorical, diary-level variable)* (ref=EMS staff or managers) | | | |
| Non-ambulance healthcare professionals | 4.93 (2.93, 8.29), p<0.001* | n/a | n/a |
| Other | 0.34 (0.06, 2.02), p=0.232 | n/a | n/a |
| Patients/relatives | 0.65 (0.35, 1.21), p=0.172 | n/a | n/a |
| **Sign** *(categorical, diary-level variable)* (ref=Mixed) | | | |
| Negative | 0.18 (0.04, 0.73), p=0.017* | n/a | n/a |
| Neutral | 0.78 (0.30, 2.07), p=0.623 | n/a | n/a |
| Positive | 1.78 (0.76, 4.17), p=0.185 | n/a | n/a |
| **Format** *(categorical, diary-level variable)* (ref=Electronic) | | | |
| Other | 0.07 (0.01, 1.06), p=0.055 | n/a | n/a |
| Verbal | 0.71 (0.35, 1.42), p=0.331 | n/a | n/a |
| Written | 0.71 (0.21, 2.33), p=0.568 | n/a | n/a |
| **Lag-time** *(continuous, diary-level variable)* | | | |
|  | 1.00 (0.99, 1.00), p=0.342 | n/a | n/a |
| **Feedback-seeking behaviour** *(binary, diary-level variable)* (ref=Unsought) | | | |
| Seeking | 3.21 (1.76, 5.87), p<0.001* | 2.81 (1.48, 5.36), p=0.002* | 3.35 (1.68, 6.69), p<0.001* |
| **Formal/informal**  *(binary, diary-level variable)* (ref=Formal) | | | |
| Informal | 0.58 (0.28, 1.19), p=0.138 | n/a | 0.51 (0.23, 1.09), p=0.083 |
| **FES** *(binary, participant-level variable)* | | | |
|  | 1.02 (0.99, 1.05), p=0.202 | n/a | 1.02 (0.99, 1.06), p=0.136 |
| **Role** *(binary, participant-level variable)* (ref=EMT) | | | |
| Paramedic | 0.92 (0.34, 2.48), p=0.870 | 0.82 (0.27, 2.46), p=0.720 | 1.18 (0.36, 3.92), p=0.789 |
| **Sex** (binary, participant-level variable) (ref=Female) | | | |
| Male | 0.69 (0.35, 1.39), p=0.300 | n/a | n/a |
| **Ethnicity** *(binary, participant-level variable)* (ref=Non-white) | | | |
| White | 5.33 (0.36, 78.2), p=0.222 | n/a | n/a |
| **Length in service** *(continuous, participant-level variable)* | | | |
|  | 1.02 (0.97, 1.07), p=0.419 | 1.01 (0.96, 1.07), p=0.617 | 1.01 (0.95, 1.07), p=0.739 |
| **Age** *(continuous, participant-level variable)* | | | |
|  | 1.03 (0.99, 1.06), p=0.120 | n/a | n/a |

Basic model - Professional development: AIC=503.6. Extended model - Professional development: AIC=455.3.

|  | **Personal wellbeing** | | |
| --- | --- | --- | --- |
|  | **Univariable analysis**  **OR (95% CI), p-value** | **Multivariable - Basic research model**  **aOR (95% CI), p-value** | **Multivariable – Extended research model**  **aOR (95% CI), p-value** |
| **Content** (*categorical, diary-level variable*) (ref = Patient outcome feedback) | | | |
| Patient experience | 1.66 (0.68, 4.03), p=0.263 | 2.52 (0.97, 6.54), p=0.058 | 2.53 (0.94, 6.85), p=0.067 |
| Clinical performance | 0.74 (0.39, 1.40), p=0.354 | 0.97 (0.49, 1.95), p=0.942 | 1.06 (0.52, 2.15), p=0.882 |
| **Source** *(categorical, diary-level variable)* (ref=EMS staff or managers) | | | |
| Non-ambulance healthcare professionals | 1.95 (0.98, 9.69), p=0.058 | n/a | n/a |
| Other | 1.08 (0.11, 10.9), p=0.950 | n/a | n/a |
| Patients/relatives | 3.36 (1.45, 7.80), p=0.005* | n/a | n/a |
| **Sign** *(categorical, diary-level variable)* (ref=Mixed) | | | |
| Negative | 0.12 (0.02, 0.56), p=0.007* | n/a | n/a |
| Neutral | 2.40 (0.97, 5.93), p=0.059 | n/a | n/a |
| Positive | 31.0 (11.4, 84.3), p<0.001* | n/a | n/a |
| **Format** *(categorical, diary-level variable)* (ref=Electronic) | | | |
| Other | 0.36 (0.03, 4.71), p=0.439 | n/a | n/a |
| Verbal | 0.85 (0.38, 1.86), p=0.674 | n/a | n/a |
| Written | 1.71 (0.33, 8.96), p=0.523 | n/a | n/a |
| **Lag-time** *(continuous, diary-level variable)* | | | |
|  | 1.00 (0.99, 1.01), p=0.770 | n/a | n/a |
| **Feedback-seeking behaviour** *(binary, diary-level variable)* (ref=Unsought) | | | |
| Seeking | 2.04 (1.04, 4.01), p=0.039* | 2.43 (1.17, 5.02), p=0.017* | 2.58 (1.19, 5.56), p=0.016* |
| **Formal/informal**  *(binary, diary-level variable)* (ref=Formal) | | | |
| Informal | 1.28 (0.61, 2.69), p=0.511 | n/a | 1.25 (0.58, 2.71), p=0.575 |
| **FES** *(binary, participant-level variable)* | | | |
|  | 1.03 (1.01, 1.06), p=0.013* | n/a | 1.04 (1.01, 1.07), p=0.020* |
| **Role** *(binary, participant-level variable)* (ref=EMT) | | | |
| Paramedic | 0.68 (0.23, 2.01), p=0.488 | 0.56 (0.17, 1.85), p=0.343 | 0.63 (0.19, 2.10), p=0.448 |
| **Sex** (binary, participant-level variable) (ref=Female) | | | |
| Male | 0.66 (0.32, 1.36), p=0.263 | n/a | n/a |
| **Ethnicity** *(binary, participant-level variable)* (ref=Non-white) | | | |
| White | 0.00 (0.00, Inf), p=0.814 | n/a | n/a |
| **Length in service** *(continuous, participant-level variable)* | | | |
|  | 1.01 (0.97, 1.06), p=0.606 | 1.01 (0.96, 1.07), p=0.668 | 1.01 (0.96, 1.06), p=0.773 |
| **Age** *(continuous, participant-level variable)* | | | |
|  | 1.01 (0.97, 1.04), p=0.673 | n/a | n/a |

Basic model - Personal wellbeing: AIC=388.02. Extended model - Personal wellbeing: AIC=348.6.

|  | **Service outcomes** | | |
| --- | --- | --- | --- |
|  | **Univariable analysis**  **OR (95% CI), p-value** | **Multivariable - Basic research model**  **aOR (95% CI), p-value** | **Multivariable – Extended research model**  **aOR (95% CI), p-value** |
| **Content** (*categorical, diary-level variable*) (ref = Patient outcome feedback) | | | |
| Patient experience | 0.26 (0.02, 3.78), p=0.325 | 2.12 (0.85, 5.28), p=0.108 | 2.14 (0.98, 4.68), p=0.058 |
| Clinical performance | 0.36 (0.02, 7.39), p=0.510 | 0.81 (0.95, 4.16), p=0.067 | 0.78 (0.44, 1.36), p=0.374 |
| **Source** *(categorical, diary-level variable)* (ref=EMS staff or managers) | | | |
| Non-ambulance healthcare professionals | 3.18 (0.08, 135.0), p=0.545 | n/a | n/a |
| Other | 10400000000 (0, Inf), p=1.00 | n/a | n/a |
| Patients/relatives | 0.29 (0.01, 6.01), p=0.423 | n/a | n/a |
| **Sign** *(categorical, diary-level variable)* (ref=Mixed) | | | |
| Negative | 150000000000 (0, Inf), p=1.00 | n/a | n/a |
| Neutral | 3300000 (0, Inf), p=0.999 | n/a | n/a |
| Positive | 17.1 (0.10, 2922), p=0.279 | n/a | n/a |
| **Format** *(categorical, diary-level variable)* (ref=Electronic) | | | |
| Other | 0.02 (0.00, 7.08), p=0.179 | n/a | n/a |
| Verbal | 1.82 (0.90, 37.0), p=0.698 | n/a | n/a |
| Written | 1.00 (0.05, 21.2), p=0.998 | n/a | n/a |
| **Lag-time** *(continuous, diary-level variable)* | | | |
|  | 1.00 (0.98, 1.04), p=0.975 | n/a | n/a |
| **Feedback-seeking behaviour** *(binary, diary-level variable)* (ref=Unsought) | | | |
| Seeking | 8.43 (0.55, 130.0), p=0.127 | 1.54 (0.91, 2.62), p=0.110 | 1.72 (0.99, 2.99), p=0.056 |
| **Formal/informal**  *(binary, diary-level variable)* (ref=Formal) | | | |
| Informal | 0.00 (0.00, Inf), p=0.689 | n/a | 0.64 (0.33, 1.25), p=0.193 |
| **FES** *(binary, participant-level variable)* | | | |
|  | 0.98 (0.77, 1.24), p=0.854 | n/a | 1.03 (1.00, 1.06), p=0.027* |
| **Role** *(binary, participant-level variable)* (ref=EMT) | | | |
| Paramedic | 0.00 (0.00, Inf), p=0.934 | 1.38 (0.57, 3.39), p=0.476 | 1.83 (0.70, 4.78), p=0.216 |
| **Sex** (binary, participant-level variable) (ref=Female) | | | |
| Male | 2.51 (0.00, 1581), p=0.779 | n/a | n/a |
| **Ethnicity** *(binary, participant-level variable)* (ref=Non-white) | | | |
| White | 0.00 (0.00, Inf), p=0.848 | n/a | n/a |
| **Length in service** *(continuous, participant-level variable)* | | | |
|  | 1.14 (0.56, 2.33), p=0.714 | 1.02 (0.97, 1.07), p=0.426 | 1.02 (0.98, 1.07), p=0.308 |
| **Age** *(continuous, participant-level variable)* | | | |
|  | 0.99 (0.73, 1.34), p=0.923 | n/a | n/a |

Basic model - Service outcomes: AIC=576.8. Extended model - Service outcomes: AIC=536.2.
