# Supplementary material for: Prevalence, predictors and outcomes of self-reported feedback for EMS professionals: a mixed-methods diary study
Source: BMC Emerg Med. 2024 Sep 13;24:165. doi: 10.1186/s12873-024-01082-y (PMC11395609; doi:10.1186/s12873-024-01082-y)
Supplement: Supplementary file 6 — Supplementary Material 6: Sensitivity analyses of the predicted likelihood of feedback efficacy [file 12873_2024_1082_MOESM6_ESM.docx]

**Additional file 6: Sensitivity analyses for the predicted likelihood of feedback efficacy**

|  | **Professional development** | | |
| --- | --- | --- | --- |
|  | **Univariable analysis**  **OR (95% CI), p-value** | **Multivariable - Basic research model**  **aOR (95% CI), p-value** | **Multivariable – Extended research model**  **aOR (95% CI), p-value** |
| **Content** (*categorical, diary-level variable*) (ref = Patient outcome feedback) | | | |
| Patient experience | 1.73 (0.87, 3.46), p=0.119 | 1.56 (0.76, 3.18), p=0.222 | 1.50 (0.73, 3.07), p=0.273 |
| Clinical performance | 1.07 (0.64, 1.79), p=0.806 | 1.03 (0.60, 1.76), p=0.912 | 1.02 (0.59, 1.76), p=0.942 |
| **Source** *(categorical, diary-level variable)* (ref=EMS staff or managers) | | | |
| Non-ambulance healthcare professionals | 2.03 (1.09, 3.77), p=0.025* | n/a | n/a |
| Other | 0.34 (0.06, 2.02), p=0.264 | n/a | n/a |
| Patients/relatives | 0.65 (0.35, 1.21), p=0.517 | n/a | n/a |
| **Sign** *(categorical, diary-level variable)* (ref=Mixed) | | | |
| Negative | 0.11 (0.03, 0.43), p=0.001 | n/a | n/a |
| Neutral | 0.70 (0.26, 1.85), p=0.471 | n/a | n/a |
| Positive | 1.70 (0.71, 4.06), p=0.229 | n/a | n/a |
| **Format** *(categorical, diary-level variable)* (ref=Electronic) | | | |
| Other | 0.10 (0.01, 1.29), p=0.077 | n/a | n/a |
| Verbal | 0.78 (0.39, 1.55), p=0.472 | n/a | n/a |
| Written | 0.85 (0.26, 2.74), p=0.783 | n/a | n/a |
| **Lag-time** *(continuous, diary-level variable)* | | | |
|  | 1.00 (0.99, 1.00), p=0.270 | n/a | n/a |
| **Feedback-seeking behaviour** *(binary, diary-level variable)* (ref=Unsought) | | | |
| Seeking | 3.40 (1.85, 6.22), p<0.001* | 3.29 (1.79, 6.04), p<0.001* | 3.53 (1.89, 6.60), p<0.001* |
| **Formal/informal**  *(binary, diary-level variable)* (ref=Formal) | | | |
| Informal | 0.94 (0.50, 1.77), p=0.843 | n/a | 0.74 (0.38, 1.46), p=0.389 |
| **FES** *(binary, participant-level variable)* | | | |
|  | 1.02 (1.00, 1.04), p=0.120 | n/a | 1.02 (1.00, 1.05), p=0.091 |
| **Role** *(binary, participant-level variable)* (ref=EMT) | | | |
| Paramedic | 0.88 (0.35, 2.22), p=0.786 | 0.74 (0.26, 2.10), p=0.576 | 0.81 (0.28, 2.33), p=0.694 |
| **Sex** (binary, participant-level variable) (ref=Female) | | | |
| Male | 0.86 (0.46, 1.61), p=0.641 | n/a | n/a |
| **Ethnicity** *(binary, participant-level variable)* (ref=Non-white) | | | |
| White | 2.45 (0.13, 44.7), p=0.546 | n/a | n/a |
| **Length in service** *(continuous, participant-level variable)* | | | |
|  | 1.02 (0.98, 1.06), p=0.427 | 1.01 (0.96, 1.06), p=0.625 | 1.02 (0.97, 1.07), p=0.461 |
| **Age** *(continuous, participant-level variable)* | | | |
|  | 1.02 (0.99, 1.06), p=0.142 | n/a | n/a |

|  | **Personal wellbeing** | | |
| --- | --- | --- | --- |
|  | **Univariable analysis**  **OR (95% CI), p-value** | **Multivariable - Basic research model**  **aOR (95% CI), p-value** | **Multivariable – Extended research model**  **aOR (95% CI), p-value** |
| **Content** (*categorical, diary-level variable*) (ref = Patient outcome feedback) | | | |
| Patient experience | 0.72 (0.35, 1.49), p=0.371 | 0.62 (0.29, 1.33), p=0.219 | 0.61 (0.29, 1.30), p=0.200 |
| Clinical performance | 0.66 (0.35, 1.23), p=0.188 | 0.62 (0.33, 1.17), p=0.141 | 0.62 (0.33, 1.17), p=0.138 |
| **Source** *(categorical, diary-level variable)* (ref=EMS staff or managers) | | | |
| Non-ambulance healthcare professionals | 1.90 (1.00, 3.62), p=0.049* | n/a | n/a |
| Other | 1.13 (0.12, 11.05), p=0.918 | n/a | n/a |
| Patients/relatives | 3.49 (1.53, 7.94), p=0.003* | n/a | n/a |
| **Sign** *(categorical, diary-level variable)* (ref=Mixed) | | | |
| Negative | 0.14 (0.03, 0.62), p=0.010* | n/a | n/a |
| Neutral | 2.37 (0.99, 5.64), p=0.052 | n/a | n/a |
| Positive | 26.52 (10.47, 67.17), p<0.001* | n/a | n/a |
| **Format** *(categorical, diary-level variable)* (ref=Electronic) | | | |
| Other | 0.14 (0.03, 0.62), p=0.010* | n/a | n/a |
| Verbal | 1.10 (0.99, 5.64), p=0.052 | n/a | n/a |
| Written | 1.74 (10.47, 67.17), p<0.001* | n/a | n/a |
| **Lag-time** *(continuous, diary-level variable)* | | | |
|  | 1.00 (0.99, 1.01), p=0.706 | n/a | n/a |
| **Feedback-seeking behaviour** *(binary, diary-level variable)* (ref=Unsought) | | | |
| Seeking | 2.21 (1.16, 4.22), p=0.017* | 2.33 (1.18, 4.57), p=0.015* | 2.23 (1.13, 4.40), p=0.020* |
| **Formal/informal**  *(binary, diary-level variable)* (ref=Formal) | | | |
| Informal | 1.40 (0.71, 2.75), p=0.328 | n/a | 1.17 (0.57, 2.37), p=0.672 |
| **FES** *(binary, participant-level variable)* | | | |
|  | 1.03 (1.00, 1.06), p=0.024* | n/a | 1.03 (1.00, 1.06), p=0.023* |
| **Role** *(binary, participant-level variable)* (ref=EMT) | | | |
| Paramedic | 0.78 (0.29, 2.10), p=0.616 | 0.69 (0.23, 2.10), p=0.518 | 0.82 (0.28, 2.40), p=0.717 |
| **Sex** (binary, participant-level variable) (ref=Female) | | | |
| Male | 0.77 (0.40, 1.51), p=0.451 | n/a | n/a |
| **Ethnicity** *(binary, participant-level variable)* (ref=Non-white) | | | |
| White | 0.00 (0.00, Inf), p=0.825 | n/a | n/a |
| **Length in service** *(continuous, participant-level variable)* | | | |
|  | 1.02 (0.97, 1.07), p=0.482 | 1.01 (0.96, 1.07), p=0.611 | 1.02 (0.97, 1.07), p=0.470 |
| **Age** *(continuous, participant-level variable)* | | | |
|  | 1.01 (0.97, 1.04), p=0.759 | n/a | n/a |

|  | **Service outcomes** | | |
| --- | --- | --- | --- |
|  | **Univariable analysis**  **OR (95% CI), p-value** | **Multivariable - Basic research model**  **aOR (95% CI), p-value** | **Multivariable – Extended research model**  **aOR (95% CI), p-value** |
| **Content** (*categorical, diary-level variable*) (ref = Patient outcome feedback) | | | |
| Patient experience | 1.08 (0.58, 2.04), p=0.811 | 1.02 (0.54, 1.92), p=0.964 | 0.98 (0.52, 1.87), p=0.956 |
| Clinical performance | 0.96 (0.58, 1.61), p=0.881 | 0.94 (0.56, 1.57), p=0.803 | 0.93 (0.55, 1.55), p=0.775 |
| **Source** *(categorical, diary-level variable)* (ref=EMS staff or managers) | | | |
| Non-ambulance healthcare professionals | 1.82 (1.05, 3.15), p=0.033* | n/a | n/a |
| Other | 0.46 (0.08, 2.57), p=0.375 | n/a | n/a |
| Patients/relatives | 3.67 (1.85, 7.27), p<0.001* | n/a | n/a |
| **Sign** *(categorical, diary-level variable)* (ref=Mixed) | | | |
| Negative | 0.28 (0.07, 1.13), p=0.072 | n/a | n/a |
| Neutral | 1.27 (0.51, 3.17), p=0.610 | n/a | n/a |
| Positive | 4.05 (1.80, 9.08), p<0.001* | n/a | n/a |
| **Format** *(categorical, diary-level variable)* (ref=Electronic) | | | |
| Other | 0.20 (0.02, 2.56), p=0.217 | n/a | n/a |
| Verbal | 0.94 (0.49, 1.80), p=0.860 | n/a | n/a |
| Written | 1.20 (0.38, 3.84), p=0.756 | n/a | n/a |
| **Lag-time** *(continuous, diary-level variable)* | | | |
|  | 1.00 (1.00, 1.01), p=0.980 | n/a | n/a |
| **Feedback-seeking behaviour** *(binary, diary-level variable)* (ref=Unsought) | | | |
| Seeking | 1.46 (0.88, 2.42), p=0.144 | 1.41 (0.85, 2.35), p=0.185 | 1.43 (0.85, 2.40), p=0.174 |
| **Formal/informal**  *(binary, diary-level variable)* (ref=Formal) | | | |
| Informal | 0.99 (0.53, 1.83), p=0.961 | n/a | 0.88 (0.47, 1.65), p=0.692 |
| **FES** *(binary, participant-level variable)* | | | |
|  | 1.03 (1.00, 1.05), p=0.026* | n/a | 1.03 (1.00, 1.06), p=0.023* |
| **Role** *(binary, participant-level variable)* (ref=EMT) | | | |
| Paramedic | 1.37 (0.53, 3.52), p=0.519 | 1.20 (0.45, 3.18), p=0.714 | 1.39 (0.53, 3.66), p=0.500 |
| **Sex** (binary, participant-level variable) (ref=Female) | | | |
| Male | 0.55 (0.27, 1.11), p=0.095 | n/a | n/a |
| **Ethnicity** *(binary, participant-level variable)* (ref=Non-white) | | | |
| White | 0.00 (0.00, Inf), p=0.822 | n/a | n/a |
| **Length in service** *(continuous, participant-level variable)* | | | |
|  | 1.03 (0.98, 1.08), p=0.202 | 1.03 (0.98, 1.08), p=0.298 | 1.04 (0.99, 1.09), p=0.164 |
| **Age** *(continuous, participant-level variable)* | | | |
|  | 1.04 (1.00, 1.07), p=0.041* | n/a | n/a |
